# Granulomatous Mastitis Due to Non-Tuberculous Mycobacteria: A Diagnostic and Therapeutic Dilemma

**DOI:** 10.3390/clinpract11020034

**Published:** 2021-04-14

**Authors:** Owais Ahmed Patel, Girish D. Bakhshi, Amogh R. Nadkarni, Zarin S. Rangwala

**Affiliations:** Grant Government Medical College and Sir JJ Group of Hospitals, Mumbai 400008, India; gdbakhshi@yahoo.com (G.D.B.); amoghrn@gmail.com (A.R.N.); zarinrangwala@gmail.com (Z.S.R.)

**Keywords:** breast, mastitis, non-tuberculous mycobacteria, breast abscess

## Abstract

Non-tuberculous mycobacterial (NTM) infections of the breast are rare. These infections present as cellulitis of the breast or breast abscess. Their diagnosis poses a challenge as they manifest signs of acute inflammation, unlike tuberculous mycobacterial infections which present in a chronic pattern. However, on aspiration of pus from the site of infection, primary smear may show acid fast bacilli. This poses a diagnostic dilemma. The present case is that of a 34-year-old woman who presented with recurrent mastitis. She had history of right breast swelling, for which surgical excision had been performed three months prior at another facility. Her histopathology had showed cystic granulomatous neutrophilic mastitis (CNGM). The patient again presented with right breast abscess which was confirmed on ultrasonography. Incision and drainage along with removal of necrotic tissue was done. Primary smear of pus showed acid fast bacilli on Ziehl–Neelson staining. Bacterial culture and line probe speciation revealed non-tuberculous mycobacterium *M. abscessus*, which responded well to prolonged anti-microbial therapy. These rapidly growing NTM require prolonged treatment and are quite often recurrent. *M. abscessus* is a rare cause of CNGM, with this being only the third reported case in literature. A brief case report with a review of literature is presented.

## 1. Introduction

Cystic neutrophilic granulomatous mastitis (CNGM) remains a little-known benign breast entity with obscure aetiology first reported in 1972 as a differential mimicking breast carcinoma [1]. Less than 1% of all breast specimens show CNGM. It is commonly observed in women of childbearing age, often with a history of breast feeding. It is typically associated with the gram-positive bacillus *Corynebacterium kroppenstedtii*. A mainstay of therapy in CNGM remains focused on lipophilic antibiotics and surgical excision in refractory cases. CNGM is rarely associated with non-tuberculous mycobacteria (NTM) [2]. NTM may cause infections of breast tissue after cosmetic surgery and in immunosuppressed individuals. These mycobacterial species show resistance to conventional antibiotic therapy. We hereby present a case of *Mycobacterium abscessus* associated with CNGM in India.

## 2. Case Presentation

A 34-year-old woman from Maharashtra, India presented with right breast swelling, generalized malaise, and decreased appetite. She gave prior history of bilateral breast abscesses three months back, for which she underwent incision and drainage at another facility. Histopathology reports had demonstrated acute on chronic granulomatous mastitis with the presence of Langhans’ type of giant cells with acid-fast bacilli seen on Ziehl–Neelson staining. She was started on a course of anti-tubercular drugs at the previous facility. The patient denied any history of previous trauma to the breast or diabetes mellitus. She reported no history of breastfeeding during the period when she developed the abscess.

**Figure 1:**
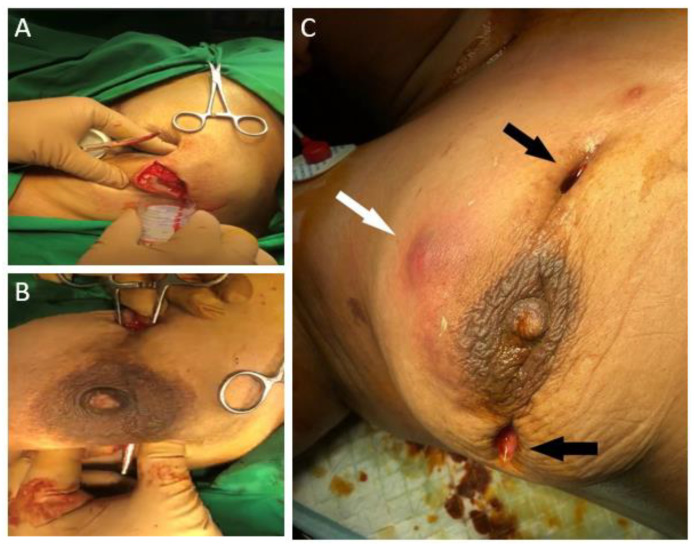
(**A**): Abscess containing pus (**B**): Communicating abscesses (**C**): Actively draining sites of previous incision (black arrows). New onset induration (white arrow).

**Figure 2:**
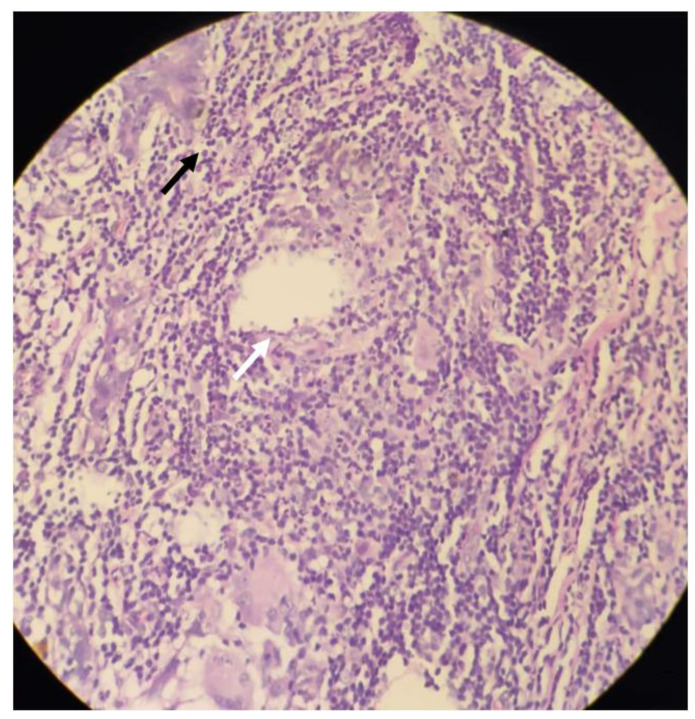
Histopathological photograph, Haematoxylin & Eosin stain, 40×; white arrow showing epithelioid cells around a granuloma; black arrow showing neutrophilic infiltrate.

At presentation, she reported a swelling on her right breast associated with throbbing pain and low-grade fever. The left breast showed a healthy scar of previous surgery. Vital parameters were unremarkable and local examination demonstrated a tender swelling over the right breast in the upper and outer aspect. It was about 10 cm × 7 cm in size and associated with local warmth, induration, and fluctuation. Blood tests revealed a leucocyte count of 18,000/mm^3^ with neutrophilia. A diagnosis of recurrent breast abscess was made. She underwent incision and drainage of the breast under general anaesthesia. Intra-operatively an indurated mass with multiple small abscesses containing greyish green pus was seen (Figure 1A,B). About 150 cc of pus was evacuated and all necrotic tissue was debrided. This pus cavity was in continuity with previously drained abscess. Post operatively she was started on a course of intravenous third generation cephalosporin. Pus was sent for Gram staining, Ziehl–Neelson (ZN) staining and culture. Gram staining was negative for bacteria, but primary ZN staining demonstrated acid-fast bacilli in fair number, suggestive of mycobacterial infection. Histopathology report was suggestive of periductal and perilobular inflammation comprising lymphocytes, plasma cells, polymorphs, foamy macrophages, histiocytes, and occasional Langhans’ type of giant cells, along with areas of micro-abscesses and frank oedema. Further, cystic spaces surrounded by neutrophilic aggregates were noted corresponding to CNGM. No micro-calcifications or granulomas were reported (Figure 2). As per hospital protocol Cartridge-based Nucleic Acid Amplification Test for *Mycobacterium tuberculosis* (GeneXpert^®^) was ordered to rule out tuberculosis. The test was negative.

Aerobic culture obtained after two weeks revealed rapidly growing non-tuberculous mycobacteria. Line probe speciation further demonstrated *Mycobacterium abscessus* sub. *abscessus* as the causative organism. Based on histological and microbiological reporting, diagnosis of cystic neutrophilic granulomatous mastitis secondary to *Mycobacterium abscessus* was made. Patient by this time had developed a new abscess at 12 o’ clock. Previous two incision sites at 10 and 3 o’ clock positions were noted to be actively draining greenish pus (Figure 1C). On incision and drainage, all three sites were noted to be communicating with each other. The necrotic breast tissue was aggressively debrided extending up to pectoralis fascia in the superior outer aspect, following which the patient was advised regular dressing changes. Anti-tubercular therapy was stopped. Based on American Thoracic Society/Infectious Diseases Society of America guidelines and antibiotic sensitivity report, combination antimicrobial therapy involving Clarithromycin and Amikacin for two weeks was given, followed by Clarithromycin monotherapy for two months. Follow up of six months has shown her to be disease and symptom free.

## 3. Discussion

CNGM is a rare disease of breast seen in around 1% of all breast specimens. It is a distinct type of granulomatous mastitis with a peculiar histopathological pattern. There is however a lack of consensus over the definition of the disease with CNGM often used interchangeably with Idiopathic Granulomatous Mastitis (IGM) and Granulomatous Lobular Mastitis (GLM). Wu et al. [3] suggested the use of ‘Cystic Neutrophilic Granulomatous Mastitis’ over IGM and GLM and proposed to recognise CNGM as a distinct entity. The characteristic feature of the disease is the presence of lobulo-centric granulomatous mastitis with cystic spaces rimmed by neutrophils and occasionally containing Gram-positive bacilli.

*Mycobacterium abscessus* is rapidly emerging nontuberculous mycobacterium that is notorious for being resistant to standard anti-microbial therapies, thus posing therapeutic challenges. Literature review showed sixteen cases of *M. abscessus* associated with infectious mastitis, demonstrated women ranging 20–54 years (Table 1) [4,5,6,7,8,9,10,11,12,13,14,15,16,17,18]. However only two cases of CNGM were associated with *Mycobacterium abscessus.*

Typically seen in parous women of childbearing age, the disease mimics several more common conditions. CNGM presents as a breast mass with nipple inversion. Breast pain, sinus formation and abscesses may also be seen. The disease is usually unilateral. However, 8.5% patients present with bilateral disease. In the present case though, the patient had a past history of surgery for bilateral breast abscesses. However, recurrent breast abscess was seen only in the right breast.

First noted as a distinct histopathological picture of CNGM in 2002, Taylor et al. were able to identify Corynebacterium species in a cohort of 34 patients with cystic lipid filled spaces surrounded by neutrophilic aggregates [19]. *Corynebacterium kroppenstedtii* was the most common isolate. However, the exact pathogenic role of coryneforms in CNGM is unknown. The present case showed acid-fast bacilli on primary smear, however a Cartridge-based Nucleic Acid Amplification Test (GeneXpert^®^) ruled out tuberculosis of the breast, which is endemic to the region. Bacterial culture revealed rapidly growing NTM which was *M. abscessus*.

Postulated aetiologies in soft tissue infections by *M. abscessus* include direct contamination by material or water in traumatic injuries or surgical wound or colonization and dissemination in immunocompromised patients. It is known to cause infection of the breast tissue in immunocompromised patients (or patients who have undergone reconstructive breast surgery [5,6,7,8,9,12,13,16]. However, in this case there was no such history).

Ultrasonography of the disease is rarely reported in literature with the most common findings being mass, dilated ducts, oedema, and abscesses [3]. Most of these findings are assigned a Breast Imaging and Reporting Data System (BIRADS) score of 4 (suspicious of malignancy). CNGM, therefore presents many diagnostic and therapeutic challenges as it mimics invasive carcinoma [1,17]. Sonography in present case showed breast abscess with necrotic tissue. Histopathologic analysis combined with bacterial culture led to the diagnosis of CNGM secondary to non-tuberculous mycobacterium. However, carcinoma of the breast and tuberculosis must be ruled out before mainstay therapy is undertaken.

Two prominent subspecies, *M. abscessus* subsp. *abscessus* and *M.* *abscessus* subsp. massiliense, have been known to encode different erm-41 gene patterns, which encodes for macrolide resistance. This poses obvious challenges as the recommended guidelines suggest Clarithromycin as the gold standard of monotherapy combined with Amikacin or Cefoxitin. Resistance rates of Clarithromycin ranges up to 20%, while Cefoxitin and Amikacin yield around 10% and 10% respectively [20]. In the present case, initially drainage of abscess and debridement of necrotic tissue was done. This was followed by intravenous amikacin for 14 days and oral Clarithromycin tablets for 10 weeks. A recurrence of mastitis is another issue associated with *M. abscessus*. Hence, these patients require prolonged antibiotic therapy and regular follow up.

## 4. Conclusions

CNGM due to non-tuberculous mycobacteria is a rare entity. It is usually seen in immunocompromised individuals or those who undergo breast reconstruction. Sometimes, no obvious predisposing factors can be found.

In regions where tuberculosis is endemic, granulomatous inflammation of the breast is usually assumed to be tubercular in origin. It is imperative that bacterial cultures and speciation are done to rule out non-tubercular mycobacterial infection to make sure the patient does not receive inadequate or potentially harmful therapies.

## Figures and Tables

**Table 1:** Review of literature of reported cases of granulomatous mastitis secondary to *Mycobacterium abscessus*, including current case.

Case Report	Age	Predisposition	Presentation	Histopathology	Treatment	Antimicrobial	Outcome
Trupiano JK (2001) [4]	17	Nipple piercing	Breast Mass	Granulomatous inflammation with AFB	Surgical Resection	Antimicrobials not received	No recurrence
Fox LP (2004) [5]	29	Breast Augmentation Surgery	Abscess	Histiocytic and giant cell reaction, granulation, AFB	Surgical Drainage	Clarithromycin x 24 weeks, Cefoxitin x3 weeks	No recurrence
Feldman EM (2007) [6]	48	Breast Augmentation Surgery	Sinus	not performed	Surgical Drainage	Clarithromycin x 24 weeks	No recurrence
Taylor JL (2006) [7]	21	Breast Augmentation Surgery with Cystic Fibrosis on Prednisone, Azathioprine, Tacrolimus	Sinus	Not reported	Surgical Drainage	Clarithromycin, Levaquin x44 weeks	Clinical deterioration & death
Pasticci (2009) [8]	54	Autoimmune Haemolytic Anaemia on prednisone	Abscess	Chronic inflammatory reaction with giant cells with AFB	Surgical Drainage	Clarithromycin x10 weeks, Amikacin	Recurrence
Jackowe DJ (2010) [9]	44	Breast Augmentation Surgery	Sinus	Not performed	Surgical Drainage	Not reported	No recurrence
Yasar et al. (2011) [10]	38	None	Breast Mass with sinus	Not performed	Aspiration	Clarithromycin x 16 weeks, Linezolid 8 weeks	No recurrence
Urganci AU (2011) [11]	27	None	Breast Mass	Granulomatous mastitis with AFB	Surgical Drainage	Clarithromycin x6 weeks	No recurrence
Ruegg (2015) [12]	39	Breast Augmentation Surgery	Abscess	Not performed	Surgical Drainage	Clarithromycin x20 weeks, Tigecycline, Linezolid, Amikacin	No recurrence
Baroudi el at. (2016) [13]	50	Crohn’s disease, off treatment	Abscess	Micro abscesses with mastitis	Antimicrobials	Clarithromycin x 12 weeks	No recurrence
Wankhade AB (2017) [14]	30	None	Breast Mass	Chronic Granulomatous mastitis	Surgical resection	Rifampin, Isoniazid, Pyrazinamide, Ethambutol, Clarithromycin, duration unknown	no follow up
Wang YS (2017) [15]	29	None	Abscess	CNGM	Surgical Drainage	Rifampin, Isoniazid, Pyrazinamide	No recurrence
Jensen et al. (2018) [16]	36	Breast Augmentation Surgery	Sinus	Not performed	Antimicrobials	Cefalexin x 8 weeks	Recurrence
Ramchandra S (2019) [17]	33	None	Breast Mass	CNGM	Antimicrobials	Clarithromycin, duration unknown	no follow up
Shaikh A (2020) [18]	32	None	Breast Mass	Mixed inflammatory infiltrate with granulomatous reaction with fat necrosis	Surgical resection	Clarithromycin x4 weeks, Amikacin 4 weeks	no follow up
Present Case	34	None	Abscess	CNGM	Surgical Drainage	Clarithromycin, Amikacin x 8 weeks	No recurrence

NR: Not reported.

## Data Availability

Data is contained within the article.
